# A New Catalogue and Insights into the 2022 Adriatic Offshore Seismic Sequence Using a Machine Learning-Based Procedure

**DOI:** 10.3390/s25010082

**Published:** 2024-12-26

**Authors:** Antonio Costanzo

**Affiliations:** Istituto Nazionale di Geofisica e Vulcanologia, 00143 Rome, Italy; antonio.costanzo@ingv.it

**Keywords:** earthquake catalogue, Adriatic offshore, seismic sequence, machine learning procedure, automatic picking, PhaseNet

## Abstract

This paper presents a new catalogue of the 2022/2023 Adriatic Offshore Seismic Sequence obtained by machine learning-based processing. The procedure performs the automatic picking and association of phases starting from the analysis of the continuous waveforms recorded by 40 seismic stations of the Italian National Seismic Network and 5 stations of the SISMIKO emergency group network. The earthquakes were detected over a 3-month period, between 1 November 2022 and 31 January 2023. This new catalogue consists of 2780 earthquakes with a magnitude equal to or greater than ML 0.4, providing more information about lower-magnitude earthquakes in particular. The results make available, on the one hand, new insights into the offshore sequence, which can contribute to confirming the attribution of the earthquakes to the Adriatic Fault System, and in particular, the mainshocks to the Cornelia fault thrust, as also hypothesised by other works in the literature. Moreover, the work provides a further contribution in showing the great potential of using machine learning-based procedures to build catalogues with a greater degree of completeness, even in very particular cases such as the one represented by the Adriatic offshore sequence, for which the minimum distance from the epicentres is high and the azimuth coverage limited.

## 1. Introduction

**Figure 1:**
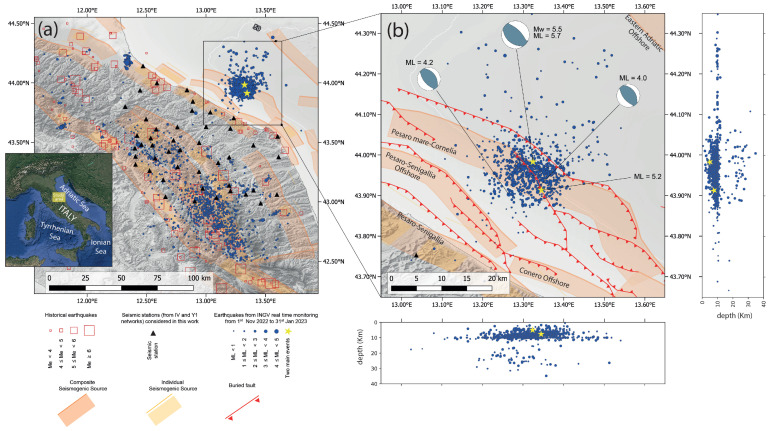
(**a**) Earthquakes localised by real-time seismic surveillance service of INGV between 1 November 2022, and 31 January 2023 (all earthquakes are available through the INGV Earthquake List at https://terremoti.ingv.it/en, accessed on 26 December 2024). The yellow stars represent the main shocks that occurred offshore in front of the Marche region coastline on 9 November 2023 (ML = 5.7 and ML = 5.2, respectively). The historical earthquakes are those reported in the CFTI5Med [30,31] and CPTI5 [32,33] seismic catalogue with the inferred equivalent magnitude (Me). In addition, the black triangle are the seismic stations belonging the permanent Italian network [9] (code IV) and the temporary INGV-SISMIKO network [8] (code Y1). The seismogenic sources are those composite and the individual ones in DISS 3.3.0. [34] (**b**) Aftershock distribution in offshore zoomed area after the mainshock of the seismic sequence. The focal mechanism for the first mainshock and for the other earthquakes with M ≥ 4 is that of TDMT [35]. The solution of the moment tensor is not available for the ML 5.2 event. Instead, the buried fault traces are from previous studies in the literature [36,37]. Hypocentres are also projected on the latitude–depth and longitude–depth section.

On 9 November 2022, a seismic sequence started off the coast of the Adriatic Sea in Italy (Figure 1a). At 06:07:25 UTC, an ML 5.7 (Mw 5.5) earthquake was detected through the real-time surveillance service operated by the National Institute of Geophysics and Volcanology (INGV). This earthquake occurred in the Marche region, approximately 27 km from the coast, at a hypocentral depth of about 5 km [1]. One minute after the main shock, an ML 5.2 earthquake was detected in the same area at a depth of 8 km [2]. The INGV control room localised 2939 earthquakes between 1 November 2022 and 31 January 2023 in the geographic area between the western part of central Italy and the offshore region of the Adriatic Sea. This area is defined by the latitude range 42.5–44.5° N and the longitude range 12.0–14.0° E in the EPSG:4326–WGS84 reference system (Figure 1a). Off the coast of the Adriatic Sea, in a zoomed area with latitudes between 43.65° N and 44.35° N and longitudes between 12.95° N and 13.65° N (Figure 1b), after the main shock, there were 751 earthquakes with magnitudes of ML ranging between 0.9 and 4.2. However, from the projection of the hypocentres in Figure 1b, it is possible to notice a sparse distribution of events at a depth of around 10 km (even very far from the mainshocks), probably due to an a priori assignment of the focal depth due to the uncertainty related to the correct localisation or to the velocity model.

After the start of this sequence, the INGV emergency groups installed and managed two temporary seismic networks: the first was deployed in the urban areas of the city of Ancona by EMERSITO [3], which was devoted to seismic site response and microzoning studies (e.g., [4,5,6,7]), whereas the other one was deployed only a few hours after the mainshock by SISMIKO [8], this network increases the sensor density of the permanent monitoring network of INGV operating on the Italian territory [9] to more reliably record the aftershocks of the sequence (e.g., [10,11]). In fact, a reliable spatial and temporal distribution of low- and moderate-magnitude earthquakes is essential to the scientific understanding of the seismic source and the progression of the sequence. However, given the offshore location of these earthquakes, it is difficult to compensate for the limited azimuth coverage in a short time. Therefore, the configuration of the network and the minimum significant distance of the seismic stations from the epicentres allowed to estimate a poorly constrained depth [12,13].

In this context, in an attempt to improve the sequence knowledge in terms of the number and location of small earthquakes, in particular, continuous waveform processing based on a machine learning method has been performed to create a new catalogue. Recently, machine learning-based techniques have been developed in many fields of earthquake seismology. The main goal of these techniques is the analysis of a large number of seismic waveforms to detect earthquakes (e.g., [14,15]), the choice of arrival time (e.g., [16,17]), phase association (e.g., [18,19]), and the location of earthquake hypocentres (e.g., [20,21]). All these steps can help improve earthquake cataloguing. However, other machine learning-based methods have been developed for quality control (e.g., [22,23]), focal mechanism analysis (e.g., [24,25]), or to investigate seismic activity based on the use of analogue records for past earthquakes (e.g., [26]). A more complete and up-to-date review of machine learning-based methods in earthquake seismology can be found in [27]. Furthermore, machine learning-based approaches have been applied in several case studies in the literature with the aim of producing high-resolution catalogues containing low-magnitude earthquakes, which can be crucial for mapping fault structures and investigating earthquake nucleation (see [28,29]). This study also aims to evaluate the benefit of employing these new methodologies, comparing the results with the information coming from the INGV catalogue, even in non-optimal conditions due to the particular configuration of the offshore network, such as the one represented by this seismic sequence.

The seismic/tectonic activity in this sector is controlled by the geodynamic behaviour of the Adria Plate; in fact, in the tectonic area affected by this seismic sequence, mild anticlines roughly orientated in the northwest–southeast direction represent the main structures of the offshore Adriatic thrust system [38,39,40]. These thrust structures are delimited to the eastern flank by reverse faults that dip between the northeast and east–northeast directions and belong to the Adriatic–Alpine–Dinaric orogenic system [12]. Therefore, the Adriatic offshore thrusts are orientated toward the southwest, in front of the central Apennines extensional fault system orogenic belt. Some authors argue that the retreat of the slab cannot be the geodynamic driving force of the northern portion of the Adriatic, and the subduction in this area beneath the Apennines ceased at least in the Early Pliocene [41]. Nevertheless, the understanding of the geodynamic activity still does not seem completely resolved, especially for the central and southern Adriatic. In fact, the different sectors are separated between them by relevant tectonic lineaments. However, most of the studies available in the literature describe the continental subduction of the lithosphere of the Adria Plate below the Apennine belt with the retreat of the slab [42,43,44,45,46], thus generating the complex tectonic framework of the area. The seismogenic character of the offshore thrust systems and the present activity have long been debated, also because of the weak geological and geomorphological onshore and bathymetric offshore characterisations, as well as the focal mechanisms of small earthquakes. Although these buried and blind offshore thrusts make it more complicated to establish their geometry and assess their activity, it is possible to state with some certainty that these are potential sources of significant earthquakes [36,37,47,48]. Moreover, historical earthquake catalogues report the localisation and effects of Mw >= 5.5 earthquakes, which have struck the Marche coast and offshore [31,32] (cf. Figure 1a). An earthquake occurred in the August 1303, with a probable offshore epicentral location, that produced tremors which were felt both in the inhabited centres of the Italian coast, with a site intensity of VIII degree of the Mercalli–Cancani–Sieberg (MCS) [49], and in Dalmatia, with an MCS intensity IX; instead, all the other historical earthquakes were located on the mainland, although very close to the coastline, producing maximum MCS intensities of up to IX [31]. A multilayer seismostratigraphic velocity model for the area of the seismic sequence was provided by Maesano et al. [36] to interpret some seismic reflection profiles collected in the VIDEPI database (visibility of oil exploration data in Italy) [50]. These profiles were collected from an offshore area in front of the Marche coast between Ancona and Pesaro, crossing the epicentral area of the seismic sequence. The stratigraphic profile includes an upper layer of Pleistocene deposits lying on the Pliocene deposits, which keep the anticlines hidden offshore. The velocity profile indicates a stratigraphic succession characterised by an increase in velocity with depth; however, this increase appears to be significantly greater for some relatively small thicknesses. Furthermore, the sonic log analysis of deep drilled wells performed by the ENI S.R.L. in recent years has allowed the interpretation of the velocity distribution along Pleistocene and Pliocene deposits in the area of the seismic sequence, revealing a rapid increase in P-wave velocity from 1.6 to 6.2 km/s, with a high-profile discontinuity between depths of 2 and 3.5 km [51,52]. This discontinuity probably corresponds to the top of carbonate rocks (named the Scaglia Formation), with velocities increasing from approximately 3.2 to 5.5 km/s within a few hundred metres. Similarly, this significant variation can be observed on the density profile; in fact, after an almost linear trend with a depth from 2.0 g/cm^3^ to approximately 2.4 g/cm^3^, the value quickly increases to 2.7 g/cm^3^ at the top of the carbonate rock [51].

## 2. Materials and Methods

The steps to obtain the new catalogue based on a machine learning method are described below.

In a first step, a Python script collected data by querying the EIDA FDNS web service [53] implemented in the ObsPy framework [54,55]. The following parameters were defined to select the waveforms to be included in the database:

- Start time on 1 November 2022 and end time on 31 January 2023;
- Spatial position of the recording stations in the area identified by a latitude between 42.98° N and 44.98° N and a longitude between 12.32° E and 14.32° E in the EPSG:4326-WGS84 reference system, i.e., ±1° of latitude and longitude of the first event;
- Only channels of seismometers (i.e., HH and EH).

The waveforms were stored in a MiniSEED format and sampled at 100 Hz. No checks were performed on the waveform data. In fact, the deep neural network of the seismic time of arrival selector is designed to calculate the probability distribution for P-waves, S-waves, and noise, and it was trained with target probability distributions of known earthquake waveforms, without applying any de-noising processes on them. Therefore, PhaseNet does not require de-noising pre-processing on the waveform data because it can recognise the characteristics of P-waves and S-waves, but it also learns what kinds of data constitute noise [56]. However, a high-pass filter characterised by a corner frequency of 1Hz and 4 corners was applied to the time histories, and this filtering improves performance for collecting events characterised by a low signal-to-noise ratio (cf. [57,58]). By scanning all waveforms in the temporary database, the arrival times of P and S waves were detected by the PhaseNet deep neural network picker implemented in Python using the standard model [56]. The threshold of the probability distribution was set to 0.5 for both the P- and S-phases, which is a value capable of ensuring the proper operation of the piker as proposed by other studies in the literature [56,59,60,61]. A total of 422,833 P-picks and 379,066 S-picks were obtained.

These P and S picks were aggregated through the Gaussian mixture model association (GaMMA) [62] to detect the phases associated with a potential earthquake, as well as its preliminary location with a homogeneous stratigraphic model characterised by a velocity of P waves of 6 km/s and a ratio between the velocity of P and S waves of 1.80. In this step, an event is detected if more than six picks are associated. This processing detected 123,699 P-wave phases and 108,751 S-wave phases associated with 17,361 events. Subsequently, the relocation of the aftershocks and mainshocks was performed using the NonLinLoc (NLL) grid search software [63], which is based on a probabilistic method [64] and a robust inversion scheme. NLL allows for the estimation of uncertainties through a probability density function, and the optimal earthquake hypocentre location is the maximum likelihood point of the computed function. Inversion was performed over an area of 110 × 110 km^2^, centred on the epicentre of the first main shock. All earthquakes detected by GaMMA, with at least eight associated phases, were relocated. The velocity model adopted in grid search processing (cf. Table 1) was obtained by combining information about the first 3 km [51], for which data from surveys in deep wells are available, and using the values provided by Di Stefano and Ciaccio (2020) [65] for greater depth. The local magnitude was obtained through a relationship proposed by Di Bona [66].

### Dataset and Codes

In summary, dataset and codes used for generating the catalogue and processing data for the figures are listed below.

- EIDA for downloading all waveforms:
  (https://www.orfeus-eu.org/data/eida/) (accessed on 22 December 2024)
- Miniconda that provides package, dependency and environment management for Python (https://www.python.org/) (accessed on 22 December 2024):
  (https://docs.anaconda.com/free/miniconda/) (accessed on 22 December 2024)
- Obspy Python framework for obtaining and pre-processing data:
  (https://docs.obspy.org/) (accessed on 22 December 2024)
- PhaseNet for detection of arrival times:
  (https://github.com/AI4EPS/PhaseNet) (accessed on 22 December 2024)
- GaMMA for association of the phases:
  (https://github.com/AI4EPS/GAMMA) (accessed on 22 December 2024)
- NonLinLoc for relocation of the detected earthquakes:
  (http://alomax.free.fr/nlloc/) (accessed on 22 December 2024)
- QGIS for producing maps in the figures:
  (https://qgis.org/it/site/about/index.html) (accessed on 22 December 2024)
- Seaborn and Pygmt Python wrappers for producing charts and cross-section in the figures:
  (https://seaborn.pydata.org/index.html) (accessed on 22 December 2024)
  (https://www.pygmt.org/latest/) (accessed on 22 December 2024)

## 3. Results

**Figure 2:**
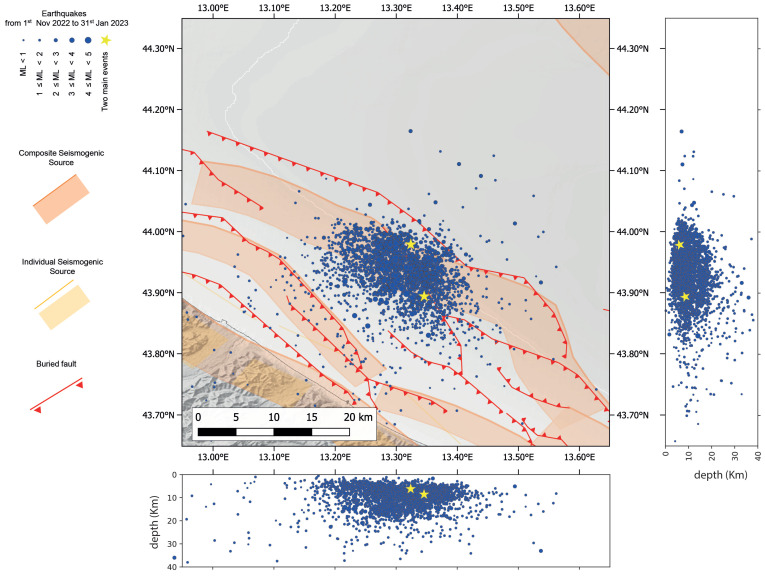
Earthquakes localised in the Adriatic offshore by machine learning-based procedure, as used in this study, between 1 November 2022, and 31 January 2023. The events were also relocated using the NonLinLoc (NLL) grid search software [63] with a 1D velocity profile representative for the area. The yellow stars are the offshore main shocks that occurred in front of the Marche region coastline (ML = 5.5 and ML = 5.2, respectively). The seismogenic sources are those composite and the individual ones in DISS 3.3.0. [34]. The buried fault traces are from previous studies in the literature [36,37]. Hypocentres are also projected on the latitude–depth and longitude–depth section.

By processing the recorded waves through the machine learning-based procedure described in the previous section, a subcatalogue of 2780 earthquakes was built for the area concerned by the seismic sequence, i.e., those events occurred in the offshore area and close to the coastline at latitudes between 43.65° N and 44.35° N and longitudes between 12.95° N and 13.65° N (Figure 2). The figure shows a clustering of offshore events and most of the earthquakes characterised by depths between 2 and 20 km. The two main events (yellow stars in Figure 2) are detected in a position very close to that provided by the INGV catalogue. The distance between the epicentres is 0.44 km for the first of the two main earthquakes and 2.12 km for the other, and they are characterised by hypocentres of about 1 km and 0.5 km deeper, respectively. Furthermore, the median local magnitudes are equal to or slightly lower than those of the INGV catalogue; in fact, the magnitudes are 5.5 for the first mainshock, and 5.2 for the second, compared to 5.7 and 5.2.

**Figure 3:**
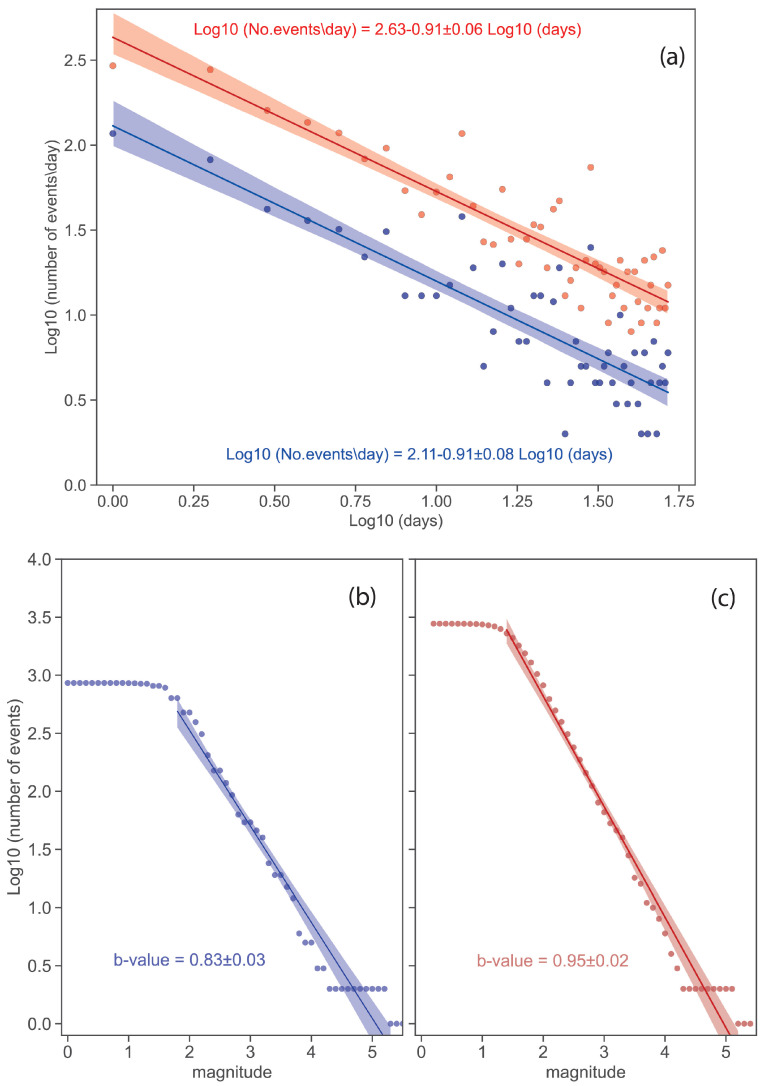
(**a**) Number of earthquakes per day as a function of the time elapsed since the mainshock from both the INGV catalogue (blue circles) and the new one based on the machine learning method (red circles). The curves represent the fitted trend for the two datasets. (**b**) Aftershock distribution following the Gutenberg–Richter relationship for the INGV catalogue with the inferred magnitude of completeness (Mc) of 1.9 and b-value 0.83. (**c**) Aftershocks distribution following the Gutenberg–Richter relationship for the new catalogue with an inferred magnitude of completeness (Mc) of 1.2 and a b-value 0.95.

In Figure 3, an overall comparison was made between the machine learning-based catalogue and that obtained by the INGV monitoring service. Figure 3a shows the daily number of events as a function of the days elapsed since 9 November 2022, i.e., when the mainshock of the Adriatic offshore sequence occurred. The interpolation curves, which represent the trends over time, indicate that the machine learning-based method detected the highest number of events. In particular, the two curves had similar slopes; therefore, the new catalogue contains on average 3.3 times the daily events of those detected by the real-time service during the observation period. However, the number of events decreased significantly after a few days: from 372 on the first day to about 20 events after 30 days.

The number of earthquakes with respect to their magnitude following the Gutenberg–Richter (GR) law [67] is reported in Figure 3b,c for the INGV catalogue and that of this study, respectively. The completeness magnitude inferred by the charts decreased significantly from 1.9 by the INGV catalogue to 1.2 for the new catalogue. Furthermore, the b-values of the GR law for the different interpolation curves indicated an improvement in the catalogue considering the entire aftershock dataset. However, it is worth noting that earthquakes ranging from magnitude between 4.2 and 5.2 are missing in both catalogues.

**Figure 4:**
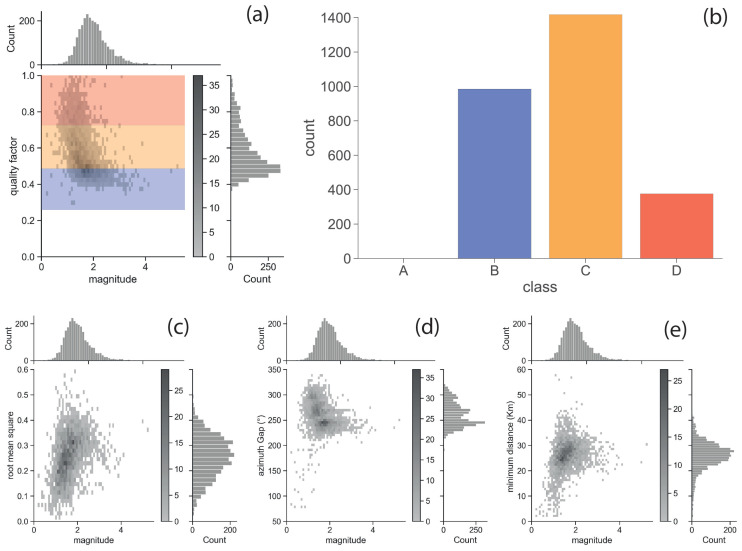
(**a**) Distribution of the quality factor in reference to the magnitude of the new catalogue following the relationship proposed by Michele et al. (2019) [68]. (**b**) Number of earthquake locations in the new catalogue falling into the four classes (from higher quality class A to a lower quality class D). Distribution of some parameters used for the quality factor: (**c**) root mean square (RMS); (**d**) azimuth gap in degree; and (**e**) distance between the epicentre and nearest seismic station in kilometres.

To classify the quality of the information regarding localisation, the factor proposed by Michele et al. (2019) [68] was calculated for the earthquakes in the offshore area (cf. Figure 4a,b). The relationship proposed by these authors returns a quality factor (q*f*) related to the position associated with the event, combining a set of uncertainty estimators (such as the root mean square, the number of phases, the azimuthal angle gap, and the errors in the hypocentral coordinates related to the covariance matrix), which are expressed in normalised form. Furthermore, the authors propose to assign a classification to the earthquake locations for a more intuitive representation following these correspondences:

- q*f* < 0.25 to class A;
- 0.25 ≤ q*f* < 0.50 to class B;
- 0.50 ≤ q*f* < 0.75 to class C;
- q*f* ≥ 0.75 to class D.

The figure shows a lower threshold for the quality factor of approximately 0.3 (cf. Figure 4a); therefore, almost all events were associated with an equal or higher value, except for two events located on the mainland closer to the coastline. It is worth pointing out that such a high lower threshold of the quality factor mostly depends on the layout of the monitoring network; in fact, although the root mean square (RMS) values were smaller than 0.3 for about 70% of the dataset (cf. Figure 4c), a limited azimuth coverage with a gap characterised by a median value greater than 255° (cf. Figure 4d) and minimum distances from the closest station of an average of 26 km (cf. Figure 4e) led to a non-negligible uncertainty in the locations of the catalogue. By assuming the ranges originally proposed by the authors [68], four different classes (from A to D) were defined to group earthquake locations from the most reliable to those with the greatest uncertainty. In this catalogue, no event was in class A, 983 were in class B, 1407 were in class C, and 390 were in class D (Figure 4b).

**Figure 5:**
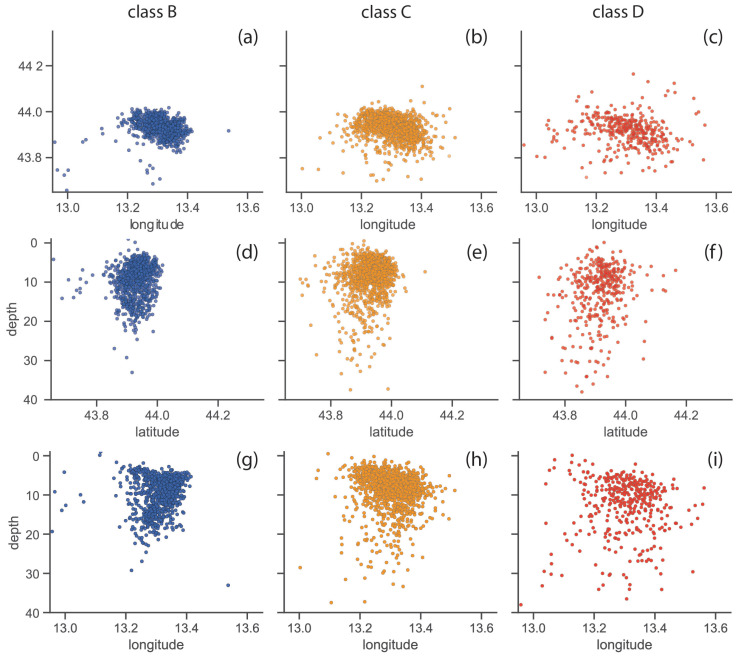
Geographic distribution of the events contained in the machine learning-based catalogue: on latitude–longitude plane for the (**a**) B-class, (**b**) C-class, and (**c**) D-class; on latitude–depth plane for the (**d**) B-class, (**e**) C-class, and (**f**) D-class; and the longitude–depth plane for the (**g**) B-class; (**h**) C-class; and (**i**) D-class.

The geographic distribution of the earthquakes in the new catalogue according to the quality classification is shown in Figure 5. All events in class B were clustered on the map (Figure 5a). As we might expect, events are more scattered in class C (Figure 5b), and even more so in class D (Figure 5c). Furthermore, observing the vertical distribution of earthquakes in classes B and C, the majority of events (approximately 90%) were located in the hypocentral depth range between 5 km and 15 km (cf. Figure 5d,g for class B and Figure 5e,h for class C), with a few others at the shallowest depths (approximately 10%) and deepest depths (approximately 14%). Of the latter events, only 17 events had depths greater than 20 km in class B, and 53 events in class C. A more distributed trend with hypocentral depth can be observed for events in class D (cf. Figure 5f,i); in fact, a much higher percentage (about 36%) of events were observed outside the depth range between 5 km and 15 km in this class. In summary, as one might expect, moving from class B to class D, it is possible to observe events less clustered in the latitude–longitude plane and, in any case, distributed over a wider depth range.

## 4. Discussion

**Figure 6:**
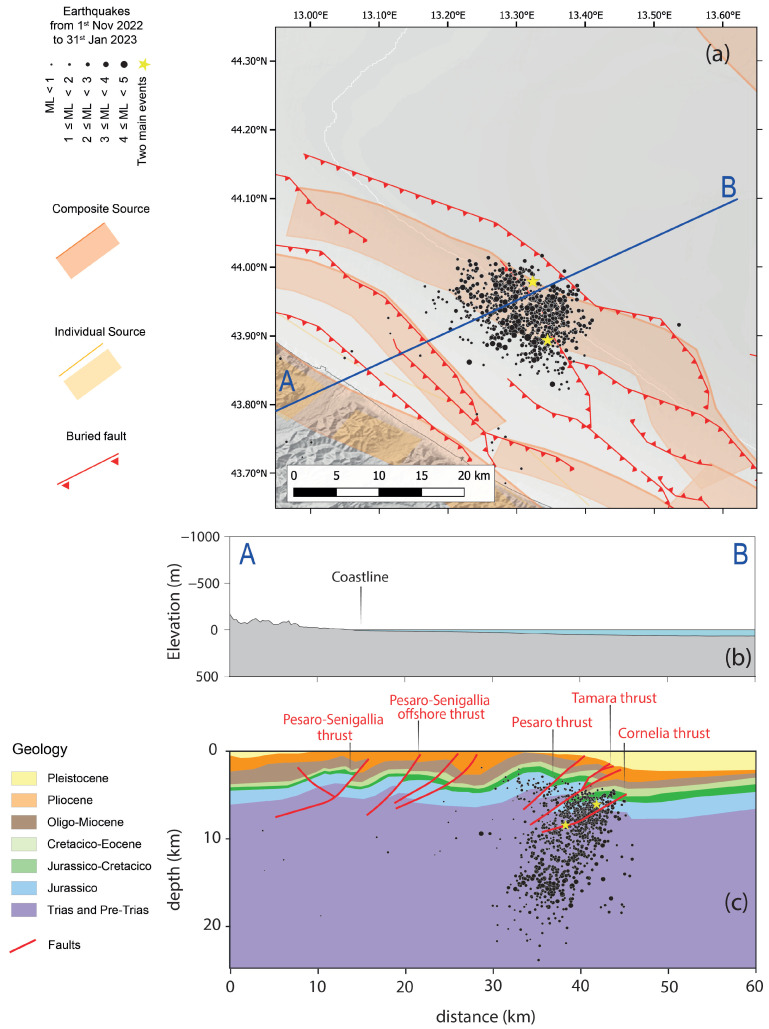
Cross-section with earthquakes in the machine learning-based catalogue and faults inferred by previous studies. (**a**) Map view with epicentres on offshore events and traces of the AB cross-section. The seismogenic sources are those composite and the individual ones in DISS 3.3.0 [34]. The buried fault traces are from previous studies in the literature [36,37]. Yellow stars are the mainshocks and the black dots are all the other events in the catalogue in the quality class B. (**b**) Sketch of the section AB, also reported in the map with an indication of the coastline. (**c**) Geological cross-section (after [36]) along AB direction. The buried faults follow the 3D tectonic model proposed by Maesano et al. [36]. The hypocentres of events in quality class B are the black dots and the mainshocks are the yellow stars, all earthquakes at a distance lower than 10 km have been projected on the cross-section.

The new catalogue allows us to make some assessments considering the distribution of events linked to the seismic sequence, also with respect to the position of the thrusts buried in the offshore area (Figure 6), which were previously deduced from geological studies and geophysical prospecting [36,38,39].

First, the events in the catalogue outline a strike in a nearly northwest–southeast direction (Figure 6a). It is worth highlighting that only the earthquakes characterised by the better quality (class B) are drawn in the figure, and the section line (Figure 6b) is also reported on the map (Figure 6a). The cross-section reported in the figure (cf. Figure 6b) with hypocentral locations and sketched faults (cf. Figure 6c) shows that events cluster along the expressions of the Adriatic thrust systems. In fact, observing the cross-section in the upper offshore area up to 10 km, most of the hypocentres are arranged along (or near) the section line representing the Cornelia thrust (cf. Figure 6c).

The two mainshocks seem to originate from the same thrust; in fact, both fall on the fault line: the first at a depth of approximately 6.2 km and the other at a depth of 8.5 km, where the fault plane has a gentler slope than the upper part. Therefore, although it is worth reiterating the uncertainty of the catalogue due to the geometry of the seismic network, the locations of most events seem to be in good agreement with previous knowledge of the earthquakes. The two mainshocks are quite confidently attributed to the Cornelia thrust in light of the insights derived from this catalogue, also confirming the findings of previous works based on geodetic observations [13] as well as geological and geophysical prospecting [36]. With reference to the 3D tectonic model proposed by [36], for which the focal mechanism of the first main event shows good consistency (cf. Figure 1b), the hypocentre of the ML 5.5 mainshock is located approximately 0.5 km above the Cornelia fault. Since this earthquake is 0.44 km away from the same event of the INGV catalogue and approximately 1.2 km deeper, the discrepancy observed by the authors themselves (hypocentre offset by approximately 1 km from the fault) is also reduced. Instead, the ML 5.2 mainshock lies just on the deeper part of the Cornelia ramp, as already founded by [36], being practically at the same depth (only 0.6 km deeper) and slightly northwest from the location in the INGV catalogue. It is worth pointing out that the solution of the moment tensor is not available for the second mainshock, since the interference of the phases of the two events did not allow it. However, focal mechanisms were calculated for other earthquakes with M ≥ 4 (cf. Figure 1b) using the time domain moment tensor technique [35].

**Figure 7:**
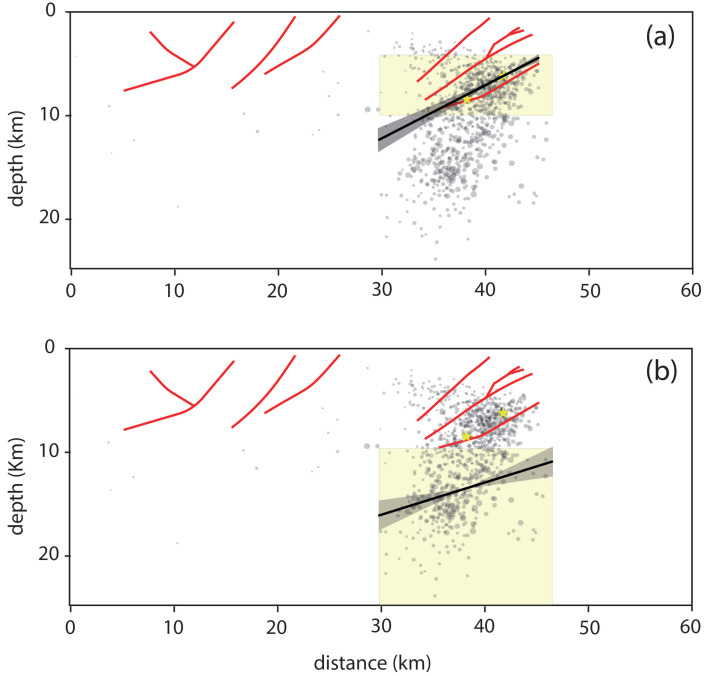
Cross-section with class B earthquakes (grey circles), the two mainshocks (yellow stars), and projected faults as inferred from previous studies (red lines). The black curves represent the least-squares fit curves to the hypocentres falling in the yellow zones: from 5 to 10 km depth (**a**) and more than 10 km (**b**). The grey area is the confidence corresponding to the standard deviation of the residuals between the obtained and estimated depths. For the shallower hypocentres (panel **a**), the curve was assumed to pass through the hypocentre of the main event.

By fitting the hypocentres around the mainshocks and with depths between 5 and 10 km with least squares regression, only assuming that the fit curve passes through the hypocentre of the first mainshock, a dip angle of about 29° was found, which closely matches the Cornelia fault of the 3D tectonic model (cf. Figure 7a).

The dip angle for the fault line is characterised by a standard deviation of 1.7 km of the residuals, which is obtained as the difference between the calculated depths and those predicted by the linear regression and corresponds to the variability of the angular estimate of about ±6°.

Furthermore, considering the events to a greater depth, a deeper fault appears, as outlined in the cross-section (cf. Figure 7b) just below the Cornelia thrust system, which could represent the upper part of the Adriatic Belt Thrust characterised by a mean dip angle of about 20°, as inferred by De Nardis et al. [40]. This fault extends to great depth up to the Moho, which is at about 40 km, as found by Di Stefano and Ciaccio [69].

As shown above, an attempt was made to fit these deeper hypocentres (depth greater than 10 km) by linear regression, thus obtaining a dip angle of 18° (cf. Figure 7b); therefore, it is lower than that obtained for the more superficial fault. However, this dip value is characterised by a more significant uncertainty; in fact, the standard deviation associated with the depth residuals is approximately 2.9 km, which corresponds to about ±10° in the angular estimation.

Finally, it is worth drawing two other broad considerations from this specific case study:

- First, only the presence of ocean bottom seismometers (OBSs) could significantly constrain the localisation of earthquakes and their depth in offshore situations such as the one under examination; in fact, despite being equipped with numerous seismic stations, the onshore network does not allow for a significant increase in the degree of uncertainty.
- Second, the use of machine learning-based re-localisation techniques, which have proven to be very promising, still seems to allow detection, especially for many more low-energy earthquakes, providing new insights for improving the knowledge and geodynamics of an area.

## 5. Conclusions

The augmented catalogue obtained by the machine learning method, which has a greater number of events than the INGV catalogue, decreases the magnitude of completeness associated with the seismic sequence, although a lack of completeness remains for magnitudes above 4.2 according to the GR law. The machine learning picking and following phase association allow for the detection of more than 17,000 earthquakes occurred in a large geographic area between the western part of Central Italy and the offshore regions of the Adriatic Sea, from 1 November 2022 to 31 January 2023. The waveforms, which were recorded by seismometers at 40 stations in the permanent network managed by INGV (network code IV) and 5 stations in the temporary network deployed by the SISMIKO emergency group (network code Y1) (cf. black triangles in Figure 1a), were processed using a machine learning method based on the PhaseNet algorithm [56] to detect arrival times. In this stage, to locate an earthquake, at least six phases must be associated.

Subsequently, a subcatalogue of 2780 earthquakes with at least eight associated phases and their closeness to the offshore area of the seismic sequence, i.e., those with epicentres with latitudes between 43.65° N and 44.35° N and longitudes between 12.95° N and 13.65° N, was extracted. Subsequently, the probabilistic approach implemented in NonLinLoc [63] was applied to this subset of data to improve localisation (Figure 2). The catalogue is reported in Supplementary Materials File S1.

The findings obtained seem to allow the attribution of the two mainshocks to one of the thrust faults belonging to the complex system of the Adriatic belt, as also found in other works available in the literature. The use of the machine learning-based technique for scanning thousands of hours of waveforms with the automatic picking function has allowed to detect many medium-low magnitude events with an improvement of the catalogue in terms of completeness magnitude, also compared to that available from the official INGV service. However, the non-optimal geometry of the network only with the stations on the mainland can significantly affect the quality of the localisation of offshore events, such as those in the sequence; in fact, for the events examined, it is not possible to go beyond class B of the localisation quality, according to the method proposed by Michele et al. (2019) [68]. The installation of monitoring stations at sea would probably be the only strategy to significantly improve data quality on the occasion of the occurrence of seismic sequences, such as the one presented in this article. However, considering the geographical conformation of Italy, the costs of installing and maintaining this type of sensor could be a significant factor to consider.

## Figures and Tables

**Table 1:** Vertical velocity profile implemented in NonLinLoc (NLL) [63] for the grid search processing: number of layers, depth, P-wave velocity ($V_{P}$), S-wave velocity ($V_{S}$), and density (ρ).

Layer	Depth (km)	$V_{P}$ (km/s)	$V_{S}$ (km/s)	ρ g/cm^3^
1	0.0	1.70	0.94	2.0
2	1.0	2.40	1.33	2.2
3	2.0	3.20	1.78	2.4
4	2.4	4.70	2.61	2.4
5	3.0	5.40	3.00	2.6
6	5.5	5.85	3.25	2.8
7	8.0	6.47	3.59	3.0
8	22.0	7.10	3.94	3.3
9	38.0	7.73	4.29	3.3
10	52.0	7.85	4.36	3.3
11	66.0	7.98	4.43	3.3
12	80.0	7.95	4.42	3.3

## Data Availability

Data are contained within the article. Instead, the seismic waveforms used in the processing are downloadable from refEIDA https://www.orfeus-eu.org/data/eida/ (accessed on 22 December 2024).

## Supplementary Materials

The following supporting information can be downloaded at: https://www.mdpi.com/article/10.3390/s25010082/s1, File S1—Catalogue of the earthquakes with epicentre in the area with latitude between 43.65° N and 44.35° N and longitude between 12.95° N and 13.65° N, from 9 November 2022 to 31 January 2023.
